# The Association of Low Blood Glucose and Low Serum Cortisol Levels in Severely Ill Children Admitted to Tertiary Referral Hospitals in Malawi: A Case-Control Study

**DOI:** 10.4269/ajtmh.21-0040

**Published:** 2021-07-19

**Authors:** Fatsani Ngwalangwa, Clifford Katumbi, Queen Dube, Josephine Langton, Tim Baker, Annika Janson, Helena Hildenwall

**Affiliations:** 1 Department of Paediatrics, University of Malawi, College of Medicine, Blantyre, Malawi;; 2 Department of Public Health, University of Malawi, College of Medicine, Blantyre, Malawi;; 3 Clinical Department, Light House Trust, Kamuzu Central Hospital, Lilongwe, Malawi;; 4 Department of Paediatrics, Queen Elizabeth Central Hospital, Blantyre, Malawi;; 5 Karolinska Institutet, Department of Global Public Health, Stockholm, Sweden;; 6 Department of Clinical Research, London School of Hygiene and Tropical Medicine, London, United Kingdom;; 7 Astrid Lindgren Children's Hospital, Karolinska University Hospital, Stockholm, Sweden;; 8 Karolinska Institutet, Department of Women's and Children's Health, Stockholm, Sweden;; 9 Karolinska Institutet, Department of Clinical Science, Intervention and Technology, Huddinge, Sweden

## Abstract

Low blood glucose concentrations < 5 mmol/L in severely ill children presenting to hospitals in low-income countries are associated with mortality. Adrenal insufficiency with low cortisol levels may contribute to low blood glucose concentrations. Understanding the association between low cortisol and low blood glucose may assist in improving guidelines for management of severely ill children. The study aimed to determine the association between low serum cortisol and low blood glucose in severely ill children. A matched case-control study of children aged 1 month to 15 years was conducted at two tertiary hospitals in Malawi. Cases were children with blood glucose <  5 mmol/L. Two age-matched controls with blood glucose of ≥  5–15 mmol/L were enrolled per case. Low cortisol was defined as serum cortisol of < 25 µg/dL (690 nmol/L) and adrenal insufficiency as serum cortisol of < 10 µg/dL (276 nmol/L). A total of 54 cases and 108 controls were enrolled with, median age of 2.8 years (interquartile range [IQR]: 1.7–4.4). The median cortisol level was 58.7 µg/dL (IQR: 42.3–61.8) in cases and 40.9 µg/dL (IQR: 33.7–51.2) in controls (*P* = 0.911). The proportion of low cortisol was 4/54 (7.4%) in cases and 9/108 (8.3%) in controls. Logistic regression shows no association between low cortisol and low blood glucose (adjusted odds ratio: 0.33; 95% confidence interval, 0.04–3.02). Results suggest that there is no association between low cortisol and low blood glucose among severely ill children presenting to hospitals in Malawi. The reason for low blood glucose needs further investigation.

## INTRODUCTION

A normal blood glucose level ensures sufficient energy supply to vital organs in the body, especially the brain.^1^ The blood glucose level is maintained by the interplay of the glucose-lowering action of insulin and the glucose-raising action of the counter-regulatory hormones cortisol, catecholamines, glucagon, and growth hormone.^2^ Low blood glucose may occur if there is an imbalance in the regulatory hormones or if there are diminished levels of glucose or its substrates in the body.^2^

Hypoglycemia is a common metabolic condition in pediatric emergencies in low-income countries.^3^ The World Health Organization (WHO) currently defines pediatric hypoglycemia as a blood glucose value < 2.5 mmol/L or < 3 mmol/L in a severely malnourished child.^4^ The prevalence of hypoglycemia at admission among African pediatric patients has been reported as up to 7.3%.^5^ Hypoglycemia may result in seizures, altered consciousness, coma,^3^ as well as increased risk of mortality.^5^^,^^6^ Studies have shown > 3-fold higher mortality also in children with “low glycemia,” which is defined as a blood glucose above the WHO cut-off of 2.5 mmol/L, with a variable upper limit of up to 5 mmol/L.^7^^–^^9^ This has led to the questioning of the current cut-off for hypoglycemia. However, a recent study by our group did not show any mortality reductions from treating low-glycemic children with intravenous dextrose.^10^

A normal response to acute illness is an increased blood glucose due to the release of cortisol in response to the acute stressor caused by the illness.^2^ However, as the acute illness progresses, the adrenal glands may become unable to release sufficient amounts of cortisol, manifesting in adrenal insufficiency and low levels of cortisol with possible consequences on glucose levels. Studies in adults have demonstrated a positive correlation between low serum cortisol, severity of illness, and an increased risk of death.^11^^,^^12^ Prevalence rates of adrenal insufficiency determined by low cortisol levels of up to 60% of critically ill adult patients with sepsis have been reported in another study.^13^ However, different cut-offs have been used to define adrenal insufficiency.^14^^,^^15^

In addition to substrate depletion, we hypothesized that low glucose levels in severe sickness were related to an exhausted stress response and low cortisol levels causing an inability to counter-regulate the low glucose and hypoglycemia. This study was conducted to determine the association between low blood glucose and low serum cortisol among severely ill children admitted to two tertiary referral hospitals in Malawi.

## METHODS

This was a case-control study of severely ill children aged 1 month to 15 years presenting to the pediatric emergency departments at Queen Elizabeth Central Hospital (QECH) and Zomba Central Hospital (ZCH) in Malawi from March 2019 to June 2020.

Queen Elizabeth Central Hospital is the largest referral hospital in Malawi, with 1,000 inpatient beds. The pediatric department serves 100,000 children a year for various illnesses, with approximately 24,000 admissions annually. All severely ill children with any WHO emergency sign^16^ are admitted via the resuscitation room of the pediatric accident and emergency unit and later transferred to the ward after stabilization. Zomba Central Hospital is a 500-bedded referral hospital, and pediatric patients are admitted from the under-five clinic or directly to the ward. Approximately 16,000 children are admitted at Zomba Central Hospital every year.^17^

The Malawi government rolled out WHO’s Emergency Triage, Assessment and Treatment guidelines for the management of severely ill children in all government hospitals in 2009.^18^ According to the standard procedures in both hospitals, children who present with any WHO emergency sign^16^ (obstructed/absent breathing, central cyanosis, coma, convulsion, shock, severe dehydration) are identified by the hospital triage nurse upon arrival to the hospital for immediate assessment, treatment, and stabilization. After triaging, all children with severe illness have their vital signs assessed, and a test for capillary blood glucose at point of care is performed. Other emergency blood samples, like malaria Rapid Diagnosis Test and Packed Cell Volume, are taken if clinically indicated.

For this study, all children 1 month to 15 years with any WHO emergency sign^16^ and a valid blood glucose result were screened by a study nurse at the pediatric emergency department for possible recruitment as soon as they had been triaged. The participants were recruited within weekday working hours from 7:30 AM to 4:30 PM. Potential participants and their guardians were enrolled after having received information about the study and provided their consent.

Cases were children with at least one WHO emergency sign and a blood glucose level of < 5.0 mmol/L at presentation. Each case was matched with two controls who also presented with at least one WHO emergency sign but had a blood glucose between 5.0 and 15.0 mmol/L. A blood glucose of up to 15.0 mmol/L was considered nonpathological because the study population was severely ill, and hyperglycemia commonly occurs in severe illness.^19^^,^^20^ Matching was done for age, accepting ±6 months difference for controls of cases below 5 years of age and ±1 year for cases from 5 years and above. Children were not included if presenting with any of the following study exclusion criteria: 1) known diagnosis of diabetes or any other glucose metabolism disorder, 2) adrenal tumor, 3) thyroid disease, 4) insulin or any other diabetic medication, 5) steroid medication, or 6) severe acute malnutrition.

All enrolled children had information collected on age, sex, WHO emergency signs at presentation, vital signs (pulse rate, respiratory rate, oxygen saturation, body temperature, and Blantyre Coma Score), fasting hours (as reported by the guardian), and whether the participant was referred from another facility. Blantyre Coma Score is a pediatric coma scaling system that was initially developed for children with cerebral malaria but is now widely used in general pediatric care.^21^ Guardians were asked questions on the child’s past medical history, previous admissions, and the presence of any chronic illness (e.g., cerebral palsy, cancer, sickle cell anemia, tuberculosis, HIV, heart disease, or congenital anomalies). A brief history of previous admissions and other illnesses was taken. The presence of any of the above-mentioned conditions or more than three admissions in the past 12 months was considered as a chronic illness.

Venous blood samples were collected for all enrolled participants immediately after enrolment, and the time of collection was documented. Cortisol samples were collected in a vacutainer blood collection tube containing spray-coated silica and a gel for serum separation. Blood was drawn using a syringe from the cannula or directly from the vein into the collecting tubes. The cortisol samples were centrifuged within 30 minutes of collection and then immediately stored in a freezer at −10°C or less until transported in a cooler box for analysis at a private laboratory outside the College of Medicine and Queen Elizabeth Central Hospital. Cortisol samples were analyzed using chemiluminescent microparticle immunoassay technology using ARCHITECT Cortisol assay (Abbot Laboratory, Abbot Park, IL)^22^ to quantitatively determine the serum cortisol. A cortisol level of < 25 μg/dL (690 nmol/L) was considered low,^14^^,^^23^ and a cortisol level < 10 μg/dL (276 nmol/L) was considered adrenal insufficiency.^15^

To calculate the sample size, the proportion of low cortisol in the controls was estimated at 20% based on a prevalence from another study conducted in patients with severe illness.^24^ The proportion in severely sick children with low blood glucose was assumed to be 45%. Using the Fleiss formula for correlation^25^ with a power of 80% and a significance level of 0.05, we calculated a required total sample of 150 children (50 cases and 100 controls).

Data were entered into Epi info version 7.4 (CDC, Atlanta, GA) and then exported to Microsoft Excel (Microsoft Corporation, Redmond, WA) for data cleaning. Stata version 14 (StataCorp, College Station, TX) was used for analysis. Basic characteristics of all the study participants were described using proportions, median and means as appropriate. Characteristics of the cases was compared with controls. A χ^2^ test was conducted to analyze if the proportion of children with low cortisol was different between cases and controls. Multivariable conditional logistic regression was conducted using the following variables selected by the investigators based on clinical plausibility: 1) sex, 2) Presence of severely deranged vital signs as defined in Table 2, 3) prolonged fasting (> 8 hours), 4) chronic illness as described in the Methods section, 5) time of blood sample collection categorized as morning (7:30 am to 12:00 pm) and afternoon (after 12 pm to 4:30 pm), and 6) whether a participant was referred from another facility. The cut-offs for determining severely deranged vital signs (Table 2) were derived from a previous study based on local and international guidelines for age specific cut-offs.^9^ Conditional logistic regression analysis of the association of cortisol and blood glucose level was also conducted.

**Table 1:** Characteristics of study participants

Variable	All (*N* = 162)	Cases (*N* = 54)	Controls (*N* = 108)
Sex, *n* (%)			
Female	61 (37.6)	18 (33.3)	43 (39.3)
Age, *n* (%)			
1 month–5 years	129 (79.6)	43 (79.6)	86 (79.6.6)
5–15 years	33 (20.4)	11 (20.4)	22 (20.3)
Median age (IQR)	2.8 (1.7–4.4)	2.8 (1.7–4.4)	2.8 (1.6–4.3)
Time of sample collection			
7:30 am–12:00 pm	82 (50.6)	29 (53.7)	53 (49.1)
> 12:00 pm–4:30 pm	80 (49.4)	25 (46.3)	55 (50.9)
One or more severely deranged vital sign,* *n* (%)			
Yes	122 (76.3)	38 (71.7)	84 (78.4)
WHO emergency signs,† *n* (%)			
Obstructed breathing	3 (1.7)	0 (0.0)	3 (2.8)
Central cyanosis	12 (7.4)	1 (1.9)	11 (10.3)
Respiratory distress	63 (38.9)	18 (33.3)	45 (41.7)
Coma	73 (45.1)	28 (51.9)	45 (41.7)
Convulsion	69 (42.6)	25 (46.3)	44 (40.7)
Shock	9 (5.6)	3 (5.6)	6 (5.6)
Severe dehydration	11 (6.8)	3 (5.6)	8 (7.4)
Fasting time, *n* (%)			
< 8 hours	80 (49.4)	22 (40.7)	58 (53.7)
≥ 8 hours	82 (50.6)	32 (59.3)	50 (46.3)
Chronic illnesses, *n* (%)			
No	151 (93.2)	52 (96.3)	99 (91.7)
Yes	11 (6.8)	2 (3.7)	9 (8.3)
Referred, *n* (%)			
No	25 (15.4)	9 (16.7)	16 (14.8)
Yes	137 (84.6)	45 (83.3)	92 (85.2)
Blood glucose level, *n* (%)			
< 2.5 mmol/L	15 (9.3)	15 (27.8)	0 (0.0)
2.5–< 5.0 mmol/L	39 (24.1)	39 (72.2)	0 (0.0)
≥ 5.0–10.0 mmol/L	94 (58.0)	0 (0.0)	94 (87.0)
> 10.0 mml/L	14 (8.6)	0 (0.0)	14 (13.0)
Median (IQR)	6.3 (4.5–8.0)	3.7 (2.3–4.5)	7.4 (6.3–8.6)

IQR = interquartile ratio.

* Age-specific cut-offs for respiratory rate, pulse rate, body temperature, Blantyre coma score, and oxygen saturation as described in Table 2.

† Participants can have more than one emergency sign; total percentage may be more than 100.

**Table 2:** Age-specific cut-offs for severely deranged vital signs

Vital sign	Age	Severely deranged
Respiratory rate, breaths/min	< 1 month	< 20 or > 80
	1 month–< 1 year	< 15 or > 60
	1–< 5 years	< 10 or > 50
	5–12 years	< 8 or > 40
	> 12 years	< 8 or > 30
Saturation, %	All	< 90
Pulse rate, beats/min	< 1 month	< 80 or > 200
	1 month–< 1 year	< 80 or > 180
	1–< 5 years	< 70 or > 170
	5–12 years	< 60 or > 150
	> 12 years	< 40 or > 130
Blantyre coma score	All	≤ 2/5
Axillary temperature, °C	< 1 month	< 35.5 or > 38.5
	≥ 1 month	< 34 or > 40

The cut-offs were derived from a previous study based on local and international guidelines for age specific cut-offs.

**Table 3:** Proportion of low cortisol among cases and controls

Variable	All (*N* = 162)	Cases (*N* = 54)	Controls (*N* = 108)	*P* value*
Cortisol levels, *n* (%)				
< 25 µg/dL	13 (8.0)	4 (7.4)	9 (8.3)	0.834
> 25 µg/dL	149 (92.0)	50 (92.6)	99 (91.6)	
Median (IQR)	43.5 (35.1–60.8)	58.9 (42.3–68.1)	40.9 (33.7–51.2)	

IQR = interquartile range.

* Chi-square *P* value.

All eligible participants and their guardians were given oral and written information about the study from the study nurse. An informed consent form was read out loud to the study participants and their guardians, which included information on the reason for conducting the study and potential harm or discomfort caused by study procedures. For all children aged 7 years and above, an assent was obtained in addition to the consent from the guardian. Participants were free to withdraw their participation in the study or use of their data at any point.

An approval to conduct the study was obtained from College of Medicine Research Ethics Committee for ethical review P.02/19/2589. Consent to conduct the study was sought from the hospitals’ directors and the head of the pediatric department at the College of Medicine.

## RESULTS

### Baseline characteristics of study participants.

In this case-control study, a total of 162 children were enrolled (54 cases; 108 controls). Table 1 gives an overview of background characteristics of included children. The median age was 2.8 years (interquartile range [IQR]: 1.7–4.4). There were in total 61/162 (37.6%) female subjects, with 18/54 (33.3%) female subjects among cases versus 43/108 (39.3%) female subjects among controls. Most of the patients (122/162; 76.3%) presented with at least one severely deranged vital sign. More than half (96/162; 59.3%) of the study participants were enrolled at Zomba hospital. Background characteristics were distributed equally between cases and controls except for fasting for more than 8 hours prior to study inclusion, which was more common among cases than controls (Table 1). The median cortisol level was 58.9 µg/dL (1,623 nmol/L; IQR, 42.3–68.1 µg/dL) in cases and 40.9 µg/dL (1,092 nmol/L; IQR: 33.7–51.2 µg/dL) in controls (*P* = 0.834) (Table 3).

### Association of serum cortisol and low glycemia.

A total of 13/162 (8.0%) patients fulfilled the diagnosis of low cortisol with a cortisol level of < 25 µg/dL (690 nmol/L). The proportion of low cortisol was 4/54 (7.4%) in cases and 9/108 (8.3%) in controls (*P* = 0.834). The proportion of low cortisol was highest among hypoglycemic patients (< 2.5 mmol/L); the proportion was 3/15 (20%) compared with 1/39 (2.6%) in low glycemia (2.5–5.0 mmol/L) and 9/108 (8.33%) in normal glycemia (5.0–15.0 mmol/L), but this was not significant (*P* = 0.105). None of the participants was diagnosed with adrenal insufficiency with serum cortisol of < 10 µg/dL (276 nmol/L).

Logistic regression showed no association between low blood glucose and low cortisol (adjusted odds ratio: 0.33; 95% CI: 0.04–3.02) (Table 4). Linear regression analysis did not show any significant change in blood glucose level with an increase in cortisol levels (β coefficient: −0.004; 95% CI: −0.020 to 0.012).

**Table 4:** The association of low cortisol with low blood glucose

Variable	UOR	*P* value	95% CI	AOR^*^	*P* value	95% CI
Cortisol levels (Ref > 25)	0.6	0.604	0.1–3.4	0.3	0.312	0.04–3.0
Severely deranged vital sign	0.7	0.518	0.3–2.0	0.7	0.495	0.2–2.0
Fasting time (Ref < 8 hours)	1.1	0.818	0.5–2.5	1.2	0.709	0.5–2.8
Sex (Ref male)	0.9	0.928	0.4–2.4	0.9	0.812	0.3–2.4
Referred	0.9	0.891	0.3–3.0	0.9	0.946	0.3–3.2
Time of the day (Ref morning)	1.1	0.761	0.5–2.7	1.2	0.672	0.5–3.3

AOR = adjusted odds ratio; CI = confidence interval; UOR = unadjusted odds ratio.

* Adjusted odds ratio for all the covariates in the table.

## DISCUSSION

In this study of severely sick children in Malawi, the proportion of low cortisol among those with low blood glucose concentrations was not different from those with normal blood glucose levels. No association was found between low serum cortisol and low blood glucose.

These findings are contrary to other studies that have shown an association between low cortisol levels and low blood glucose levels.^26^^,^^27^ Adrenal insufficiency, or low cortisol, occurs in severe illness, and some studies have reported prevalence as high as 60% among severely ill adult patients with septic shock.^13^ In this study we hypothesized that low glycemia could be a manifestation of low cortisol or adrenal insufficiency in severe illness.

Although adrenal insufficiency has reportedly been high among septic patients, studies reporting an association between low cortisol and low blood glucose levels were conducted in patients without severe illness.^26^^,^^27^ Hence, the lack of association in this study may be explained by a disruption of the normal metabolic balance where glucose variability occurs in severe illness, causing an unpredictable cortisol–glucose relationship.^20^^,^^28^^,^^29^

In the studied children, 8% fulfilled the criteria of low cortisol. However, the distribution was similar between cases and controls. These children possibly had a disruption of the hypothalamic-pituitary-adrenal axis related to the severe illness and were either not able to secrete as much cortisol in response to the stimulus triggered by severe disease or inactivated the cortisol faster.^30^ However, no child had an overt adrenal insufficiency with cortisol values of < 10 μg/dL. It may be that children with illness-induced adrenal insufficiency would not survive the initial phase of severe acute illnesses and would die before arrival to hospital because high mortality has been seen in patients with adrenal insufficiency.^31^^,^^32^

Our results suggest that the mechanisms for low or hypoglycemia are not simply explained by falling cortisol values and that more complex interactions should be considered. The reason for low glycemia in severely sick children must be sought elsewhere. It could possibly be due to fasting and poor feeding in severe illness^33^ coupled with poor reserves of glucose due to underlying moderate malnutrition,^34^ which is common in the study setting.^35^ Severe illness increases catabolic demands for glucose, diminishing body reserves of glucose in combination with a limited intake of glucose.^36^ Indeed, the proportion of fasting in this study was much higher among cases than controls and suggests that fasting plays an important role in the development of low glycemia and hypoglycemia. We also noted a higher proportion of controls had severe respiratory distress compared with cases, which may suggest that low blood glucose levels are common in certain conditions.

Conversely, 26% children had a noticeable high cortisol level (> 60 μg/dL), indicating high stress levels due to acute severe disease. The observed cortisol levels in our study were high, such that 26% children had cortisol level > 60 μg/dL. However, the values were similar to other published studies conducted in children with septic shock admitted in the intensive care unit.^37^^,^^38^ A study conducted in critically ill children found that children who developed adrenal insufficiency had higher cortisol at baseline as compared with children who did not develop adrenal insufficency.^39^

There are a number of limitations to this study. Though study participants were severely ill, one low glucose reading might not indicate whether the low glycemia was persistent. Although there were too few cases to make statistical conclusions, there was a tendency for those with hypoglycemia (blood glucose <  2.5 mmol/L) to be more likely to have low cortisol levels, and thus we may have reported different results with another definition of low glycemia than blood glucose of <  5 mmol/L. In addition, the included children had a wide range of symptoms and diagnoses, making the study population heterogeneous, which may have affected our results. Matching for the presenting WHO emergency sign would have possibly produced different results because a higher proportion of certain emergency signs has been noted in controls. Most other studies that have been conducted on low cortisol were done in patients with septic shock in an intensive care unit.^13^^,^^38^ Possible overestimation of the proportion difference used in the sample size calculation may have led to an underpowered sample size. However, a proportion of 45% was chosen because a smaller proportion was considered of less clinical significance.

## CONCLUSION

Though low cortisol was present in 8% of the study population, no association between low glycemia and low cortisol levels was seen in severely ill children admitted to tertiary referral hospitals in Malawi. The explanation for hypoglycemia among these children should be sought among alternative mechanisms.

**Figure 1.**
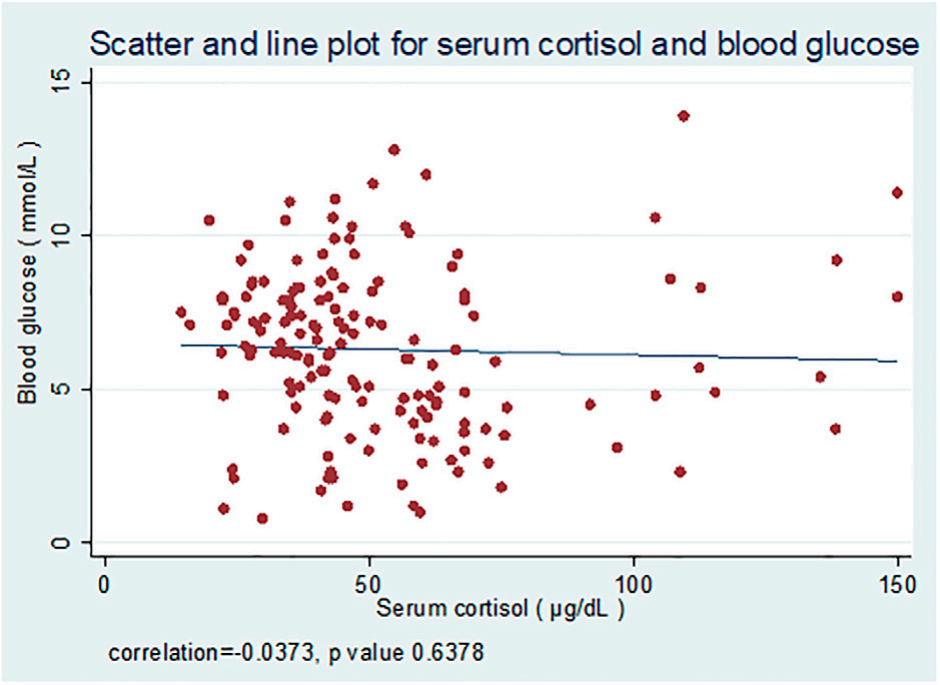
Line and scatter plot showing the relationship between cortisol levels and blood glucose levels. This figure appears in color at www.ajtmh.org.
